# Patterns of Neuropsychological Profile and Cortical Thinning in Parkinson’s Disease with Punding

**DOI:** 10.1371/journal.pone.0134468

**Published:** 2015-07-28

**Authors:** Han Soo Yoo, Hyuk Jin Yun, Seok Jong Chung, Mun Kyung Sunwoo, Jong-Min Lee, Young Ho Sohn, Phil Hyu Lee

**Affiliations:** 1 Department of Neurology and Brain Research Institute, Yonsei University College of Medicine, Seoul, South Korea; 2 Department of Biomedical Engineering, Hanyang University, Seoul, South Korea; 3 Severance Biomedical Science Institute, Seoul, South Korea; Hospital General Dr. Manuel Gea González, MEXICO

## Abstract

**Background:**

Punding, one of dopamine replacement treatment related complications, refers to aimless and stereotyped behaviors. To identify possible neural correlates of punding behavior in patients with Parkinson’s disease (PD), we investigated the patterns of cognitive profiles and cortical thinning.

**Methods:**

Of the 186 subjects with PD screened during the study period, we prospectively enrolled 10 PD patients with punding and 43 without punding on the basis of a structured interview. We performed comprehensive neuropsychological tests and voxel-based and regions-of-interest (ROIs)-based cortical thickness analysis between PD patients with and without punding.

**Results:**

The prevalence of punding in patients with PD was 5.4%. Punding behaviors were closely related to previous occupations or hobbies and showed a temporal relationship to changes of levodopa-equivalent dose (LED). Significant predisposing factors were a long duration of PD and intake of medications of PD, high total daily LED, dyskinesia, and impulse control disorder. Punding severity was correlated with LED (*p* = 0.029). The neurocognitive assessment revealed that PD patients with punding showed more severe cognitive deficits in the color Stroop task than did those without punding (*p* = 0.022). Voxel-based analysis showed that PD-punders had significant cortical thinning in the dorsolateral prefrontal area relative to controls. Additionally, ROI-based analysis revealed that cortical thinning in PD-punders relative to PD-nonpunders was localized in the prefrontal cortices, extending into orbitofrontal area.

**Conclusions:**

We demonstrated that PD patients with punding performed poorly on cognitive tasks in frontal executive functions and showed severe cortical thinning in the dorsolateral prefrontal and orbitofrontal areas. These findings suggest that prefrontal modulation may be an essential component in the development of punding behavior in patients with PD.

## Introduction

Parkinson’s disease (PD), a chronic neurodegenerative disease caused by a loss of nigral dopaminergic neurons, is characterized clinically by cardinal motor symptoms, such as bradykinesia, tremor, rigidity, and postural instability. Currently, non-motor symptoms, including psychiatric and behavioral disorders have become the focus of active concern because these symptoms can lead to a greater level of incapacity than do motor complications.[1] Today, dopamine replacement treatment (DRT)-related complications are attracting more attention. Among DRT-induced non-motor complications, impulsive control and repetitive behaviors are primarily categorized as impulse control disorders (ICDs),[2] dopamine dysregulation syndrome (DDS),[3,4] and punding behaviors.

Punding refers to aimless and stereotyped behaviors and encompasses complex, non-productive, and repetitive activities such as manipulations of technical equipment, cleaning or tidying, sorting of common objects, and hoarding. Although the exact pathophysiology of punding has not yet been established, it is suggested that neural plastic changes in the dorsal and ventral striatal structures due to chronic intermittent stimulation by dopaminergic medications and impaired reward mechanisms may be involved in a possible pathomechanism for punding.[5–7] Additionally, the current view is that the projections from the frontal cortex to the striatum inhibit the dopamine-dependent induction of the striatal stereotypies.[5] Many studies have reported that the patients with the lesion in the frontal lobe showed punding-like stereotypic behaviors.[8,9] However, no study reported so far has evaluated the relevant neurocognitive profiles, especially those involving frontal lobe functions, or the pattern of cortical atrophy. Thus, in the present study, we investigated the patterns of cognitive profiles and regional cortical thinning to identify possible neural correlates of punding behavior in patients with PD using comprehensive neuropsychological tests and voxel-based cortical thickness analysis.

## Patients and Methods

### Subjects

From March 2012 to September 2012, we prospectively enrolled 186 consecutive patients with PD at the Movement Disorder and Dementia Clinic at Yonsei University Severance Hospital who met the clinical diagnostic criteria of the UK PD Society Brain Bank.[10] We used the Minnesota Impulsive Disorders Interview and the punding rating scale developed by Fasano et al. as screening tools for ICD and punding, respectively.[11,12] All study subjects visiting the outpatient clinic during the study period answered the first question of Section I to determine the presence of punding: “Do you have any hobbies or pastimes and are you interested in them after drug (levodopa, cocaine and so on) use?” If a patient replied ‘yes’, then he/she moved on to the next step. Using the other questions in Section I, we screened those with punding from among all enrolled PD patients. If a patient fulfilled all questions in this section, we then move to Section II to evaluate the severity of punding using a rating scale. We directly interviewed all the patients or their relatives thoroughly by two neurologists (L.P.H. and Y. H.S.).

All patients complete a standardized and detailed neuropsychological test battery of the Seoul Neuropsychological Screening Battery (SNSB) to compare the cognitive function between the groups.[13] The SNSB covers attention, language, praxis, visuoconstructive function, verbal and visual memory, and frontal/executive function. We selected the study subjects whose magnetic resonance imaging (MRI) and neuropsychological data were performed within 1 year before enrollment to analyze neurocognitive and imaging data more strictly.

Of the 186 subjects with PD screened during the study period, 10 patients were identified to have punding behaviors (PD-punders). Of the remaining 176 patients without punding behavior (PD-nonpunders), 47 were excluded due to the presence of focal brain lesions or diffuse white matter hyperintensities on MRI. Another 86 patients were excluded for the followings: (a) 13 were diagnosed with PD dementia;[14] (b) 43 underwent a brain MRI or neuropsychological test more than 1 year before enrollment; (c) 18 did not had a brain MRI or neuropsychological test at all; and (d) 12 had high scores on the Beck Depression Inventory (BDI, ≥ 26 points), indicating severe depressive mood. Finally, 43 PD-nonpunders remained for further analysis. These exclusion criteria also applied to PD-punders, and none of them met the criteria. Parkinsonian motor symptoms were assessed using the Unified PD Rating Scale Part III (UPDRS-III). Total medication dose for PD was calculated in levodopa equivalents.[15] The self-rated BDI was used to assess depressive symptoms in patient with PD.[16] A [^18^F] FP-CIT positron emission tomography scan was performed on all study subjects, and all showed decreased dopamine transporter uptake in the posterior putamina. To compare the voxel-based analyses of the cortical thickness of patients with PD and age- and sex-matched healthy controls, we recruited volunteers from among elderly individuals who had been informed about the project and healthy relatives of patients with movement disorders or dementia (*n* = 23, median age = 71 yr). Members of the control group had no active neurologic disorders and no cognitive complaints with a minimal score on the Korean version of Mini-Mental State Examination (K-MMSE) of 28.

This study was approved by the Institutional Review Board of the Yonsei University Severance Hospital. Written informed consent was obtained from all patients and control subjects who participated in this study.

### MRI acquisition

All MRI scans were acquired using a Philips 3.0-T scanner (Philips Intera; Philips Medical System, Best, The Netherlands) with a SENSE head coil (SENSE factor = 2). A high-resolution T1-weighted MRI volume data set was obtained from all subjects using a three-dimensional T1-TFE sequence configured with the following acquisition parameters: axial acquisition with a 224 × 256 matrix; 256×256 reconstructed matrix with 182 slices; 220 mm field of view; 0.98 × 0.98 × 1.2 mm^3^ voxels; TE (echo time), 4.6 ms; TR (repetition time), 9.6 ms; flip angle, 8°; and slice gap, 0 mm.

### Image processing

The high-resolution T1-weighted MRI data were subjected to the following procedures, which have been described in detail elsewhere.[17–21] The data were registered to the ICBM 152 average template using a linear transformation [17], corrected for intensity nonuniformity artifacts [20], and discretely classified into white matter (WM), gray matter (GM), cerebrospinal fluid (CSF) and background using an advanced neural network classifier [21]. Hemispheric cortical surfaces were automatically extracted from each T1-weighted image using the Constrained Laplacian-based Automated Segmentation with Proximities (CLASP) algorithm, which reconstructs the inner cortical surface by deforming a spherical mesh onto the WM/GM boundary and then expanding the deformable model to the GM/CSF boundary [18, 19]. Cortical thickness was defined using the t-link method, which captures the Euclidean distance between linked vertices [22]. Each individual thickness map was transformed to a surface group template using a two-dimensional (2D) surface-based registration [23] and the mean cortical thickness of regions of interest (ROIs) using a surface-based automated anatomical labeling (AAL) template [24].

### Data analysis

We analyzed the vertex- and ROI-level differences of cortical thickness between the groups using an analysis of covariance (ANCOVA) with age, sex, PD duration, and duration of education as covariates. First, we performed the vertex-based analysis to evaluate the global structural difference and corrected *t*-statistical maps of cortical thickness. Second, in the aspect of frontal lobe, we adopted ROI-based analysis to assess the difference of cortical thickness functionally. Statistical analyses were performed using SurfStat toolbox (http://www.math.mcgill.ca/keith/surfstat/), for Matlab (R2008b; MathWorks, Natick, MA). We set the significance level for the between-group differences *p* < 0.001 in vertex-wise analysis and *p* < 0.05 in ROI-level analysis.

### Statistical analysis

Data were analyzed using the SPSS software 18.0 (SPSS, Chicago, IL, USA) and the SAS software 9.2 (SAS Institute Inc., Cary, NC, USA) for Windows. The baseline demographic characteristics and neuropsychological profiles of the two groups were analyzed and compared. The Pearson *chi*-square test or Fisher’s exact test was used to compare frequencies. The distributions of data for continuous variables were first examined for normality using Shapiro-Wilk test. When data did not deviate from normal distribution, the means and standard deviations (SDs) were calculated, and independent sample *t*-tests were applied. We used descriptive statistics, such as medians and ranges (minimum to maximum), and compared to Mann-Whitney *U* tests to examine data that were not normally distributed. Multiple linear regression analysis was used to compare the results of the neuropsychological tests, adjusting for gender, age, PD duration, and years of education, which are important considerations in interpreting neuropsychological results and evaluating frontal lobe functions. Additionally, in neuropsychological data, a composite score, dividing the sum by number of tests in each cognitive domain, was used to form reliable measures, and same statistical analysis was applied. Two-tailed *p* < 0.05 was considered to indicate statistical significance.

## Results

### Clinical characteristics of PD patients with punding behavior

Of the total 186 patients with PD who answered the questionnaire prospectively, 10 (5.4%) replied that they had punding behaviors. The mean age of PD patients with punding was 66, and 6 (60%) were male. Punding began after a median disease duration of 84 months and a median medication duration of 60 months. The median LED in punders was 655 mg per day.

**Fig 1:**
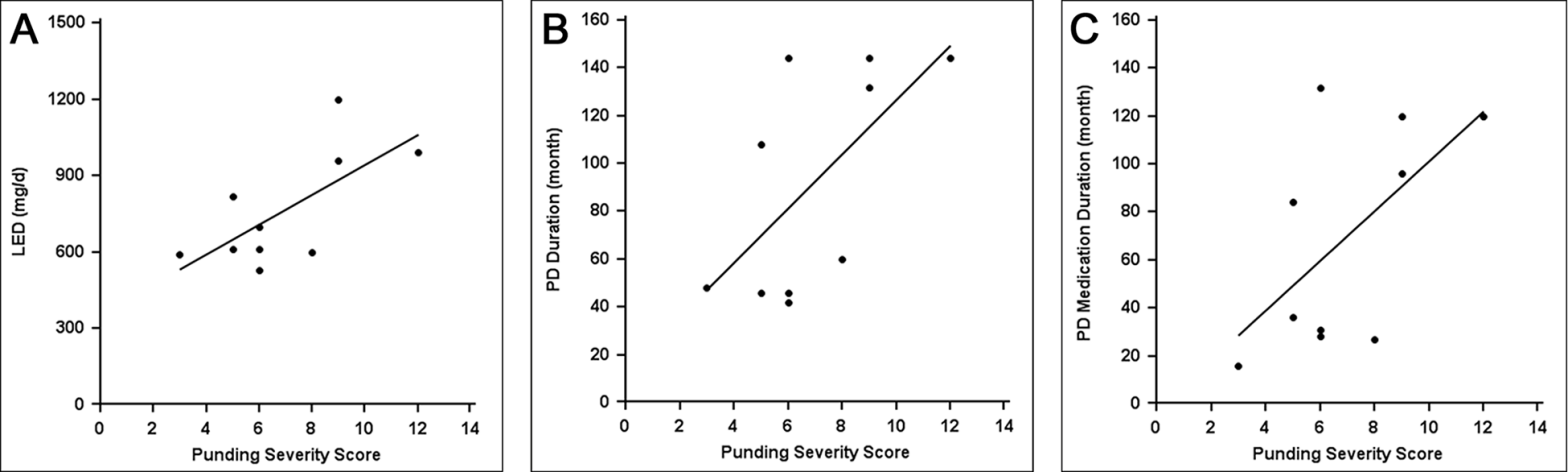
Correlation of punding severity and Parkinson’s disease (PD) duration, PD medication duration, and levodopa-equivalent dose (LED). Punding severity was significantly correlated with (A) LED (*r* = 0.69, *p* = 0.029) and showed a tendency toward an association with (B) PD duration (*r* = 0.58, *p* = 0.079). PD medication duration did not show a linear correlation with (C) punding severity (*r* = 0.52, *p* = 0.126).

The description of punding characteristics and associated symptoms in the 10 patients are presented in Table 1. In eight patients (80%), punding was closely related to a previous occupation or hobby/pastimes. Seven patients (70%) showed a temporal correlation between LED changes and the onset of punding behavior. The appearance of punding occurred within 6 months after an increase in the dose of levodopa or dopamine agonists. Interestingly, punding symptoms co-occurred in a substantial proportion of those with ICDs (60%), dyskinesia (40%), and wearing off (30%). All six patients with punding and ICDs complained of mild to severe symptoms of hypersexuality. Two patients had compulsive eating and one patient had pathologic gambling. The severity score for punding correlated significantly with the LED (*r* = 0.69, *p* = 0.029; see Fig 1A) and tended to be associated with PD duration (*r* = 0.58, *p* = 0.079; see Fig 1B).

**Table 1:** Characteristics of punding in patients with Parkinson’s disease.

Patients	Age at onset / Sex	Occupation / Hobby	Punding behavior	Hours spending on punding	Total severity score	LEG changes before the onset of punding	Associated symptoms		
							ICD	D	W
1	73/F	Housewife / Setting her house in order	Setting her house in order	8	9	Dose-up of madopar (*Δ*LED: + 150mg/d)	(-)	(+)	(+)
2	62/M	Farm work / -	Sorting out farming tools	3	3	Dose-up of stalevo and ropinirole (*Δ*LED: + 190mg/d)	(-)	(-)	(-)
3	60/M	Building contractor / Computer	Repairing things	3	5	Dose-up of ropinirole (*Δ*LED: + 40mg/d)	(-)	(-)	(-)
4	76/M	Paper manufacturing / Playing card	Internet card game (not gambling)	5	9	(-)	(-)	(+)	(+)
5	65/M	Insurance salesman / Computure	Check E-mail	5	6	Dose-up of stalevo (*Δ*LED: + 105mg/d)	HS, PG	(+)	(-)
6	72/M	High school teacher / Writing	Writing anything	3	6	Dose-up of ropinirole (*Δ*LED: + 40mg/d)	HS	(-)	(-)
7	61/F	The handicapped helper / -	Tidying and cleaning	3	8	Dose-up of ropinirole (*Δ*LED: + 60mg/d)	HS, CE	(-)	(-)
8	72/F	Housewife / Playing card	Internet card game (not gambling)	8	12	(-)	HS, CE	(+)	(+)
9	71/F	Professor in the College of Music (piano) / -	Playing the piano	4	5	Dose-up of madopar (*Δ*LED: + 80mg/d)	HS	(-)	(-)
10	56/M	Livestock industry / Baduk	Playing baduk	4	6	(-)	HS	(-)	(-)

CE = compulsive eating; D = dyskinesia; F = female; HS = hypersexuality; ICD = impulse control disorder; M = male; PG = pathologic gambling; W = wearing off; *Δ*LED = the amount of change of levodopa-equivalent dose

### Demographic characteristics of PD patients with and without punding

Table 2 presents a comparison between the basic demographic characteristics of the groups. PD patients with punding had been suffering from PD (84 vs. 36 months, *p* = 0.001) and taking PD medication (60 vs. 22 months, *p* = 0.007) for a longer period of time and their total LED (655 vs. 530 mg/day, *p* = 0.010) was higher compared with those without punding. When comparing types of antiparkinsonian medications, PD-punders tended to have a higher levodopa LED than in PD-nonpunders, whereas the two groups did not differ with regard to the dose of dopamine agonists. Additionally, PD patients with punding were given high LEDs more frequently than were those without punding. The PD-punders were also more likely to suffer from an ICD and dyskinesia compared with the PD-nonpunders (60.0% vs. 9.3%, *p* = 0.001; 40.0% vs. 11.6%, *p* = 0.044). The two groups did not differ significantly in terms of age, sex, K-MMSE, Clinical Dementia Rating, BDI, years of education, UPDRS motor scores, and the presence of wearing-off.

**Table 2:** Baseline demographic characteristics of PD patients with and without punding.

	PD-punder (*n* = 10)	PD-nonpunder (*n* = 43)	*P*-value
**Age, year**	66.8 ± 6.8	67.1 ± 6.6	0.508
**Male, n**	6 (60.0%)	25 (58.1%)	0.602
**PD duration, month**	84.0 (42, 144)	36.0 (14, 144)	0.001
**Total LED, mg/day**	655.0 (530, 1200)	530.0 (160, 1720)	0.010
** DA agonist LED, mg/day**	160.0 (80, 280)	150.0 (0, 360)	0.111
** Levodopa LED, mg/day**	495.0 (360, 1000)	450.0 (0, 1540)	0.065
** High LED (≥800 mg/day)**	4 (40.0%)	4 (9.3%)	0.033
**Medication duration, month**	60.0 (16, 132)	22.0 (7, 120)	0.007
**K-MMSE**	27.0 (23, 30)	28.0 (22, 30)	0.612
**CDR**	0.5 (0, 1)	0.5 (0, 1)	0.518
**BDI**	17.5 (2, 24)	12.0 (1, 25)	0.426
**Education year, year**	12.0 (1, 16)	9.0 (1, 16)	0.738
**UPDRS motor**	26.4 ± 10.0	26.8 ± 11.8	0.083
**ICD**	6 (60%)	4 (9.3%)	0.001
**Dyskinesia**	4 (40.0%)	5 (11.6%)	0.044
**Wearing off**	3 (30.0%)	6 (14.0%)	0.346

The values are expressed as mean (SD), number (percentage), or median (minimum-maximum).

BDI = Beck depression inventory; CDR = clinical dementia rating; DA = dopamine agonist; ICD = impulse control disorder; K-MMSE = Korean version of Mini-Mental State Examination; LED = levodopa equivalent dose; PD = Parkinson's disease; UPDRS = Unified PD Rating Scale.

### Pattern of neurocognitive profiles

The comparison of detailed neuropsychological characteristics in PD patients with and without punding is summarized in Table 3. Compared with the PD-nonpunders, PD-punders performed significantly worse in the color Stroop task (63.90 vs. 83.35, *p* = 0.022). Meanwhile, all the other items of the SNSB including attention, memory, language, visuospatial functions did not significantly differ between the two groups. By analyzing composite score between the two groups, PD-punders exhibited a tendency of poorer performance in frontal executive functions relative to PD-nonpunders (*p* = 0.086). The composite scores in other functional domains did not show significant difference between the two groups.

**Table 3:** Neuropsychological data between PD patients with and without punding.

	PD-punder (*n* = 10)	PD-nonpunder (*n* = 43)	*P*-value
**Attention**	21.0 (14, 26)	20.0 (10, 26)	0.583^a^
Digit span (forward)	7.0 (5, 9)	6.0 (3, 9)	0.416
Digit span (backward)	4.0 (2, 5)	4.0 (0, 5)	0.904
Digit span total	10.5 (7, 13)	10.0 (5, 13)	0.612
**Language and related function**	67.5 (52, 84)	71 (38, 84)	0.652^a^
K-BNT	40.20 ± 9.47	42.23 ± 11.06	0.786
Repetition	15.0 (9, 15)	15.0 (11, 15)	0.433
Calculation	12.0 (10, 12)	12.0 (7, 12)	0.937
**Visuospatial function**	29.75 (16.0, 36.0)	34.0 (17.5, 36.0)	0.382^a^
RCFT	29.75 (16.0, 36.0)	34.0 (17.5, 36.0)	0.382
**Verbal memory function (SVLT)**	45.30 ± 8.18	43.40 ± 10.16	0.536^a^
Immediate recall	19.40 ± 4.03	18.30 ± 5.40	0.672
Delayed recall	6.5 (1, 10)	5.0 (0, 10)	0.766
Recognition	20.5 (16, 23)	20.0 (12, 24)	0.714
**Visual memory function (RCFT)**	38 (31, 76)	46 (17.5, 88)	0.413
Immediate recall	12.80 ± 7.32	14.91 ± 7.14	0.324
Delayed recall	12.00 ± 6.48	14.50 ± 7.09	0.215
Recognition	20.0 (18, 23)	19.0 (13, 22)	0.945
**Frontal executive function**	255.10 ± 48.89	286.14 ± 40.02	0.086^a^
Contrasting program	20.0 (18, 20)	20.0 (13, 20)	0.414
Go-no-go test	20.0 (18, 20)	19.0 (6, 20)	0.325
Semantic generative naming	27.50 (16, 40)	28.00 (16, 59)	0.459
Phonemic generative naming	24.80 ± 10.95	23.47 ± 11.98	0.968
Word Stroop test	112 (29, 112)	112 (79, 112)	0.197
Color Stroop test	63.90 ± 31.96	83.35 ± 18.72	0.022

The values are expressed as mean (SD), number (percentage), or median (minimum-maximum).

Data are adjusted for gender, age, PD duration, and years of education.

K-BNT = Korean Version of the Boston Naming Test; PD = Parkinson's disease; RCFT = Rey Complex Figure Test; SVLT = Seoul Verbal Learning Test.

^a^ Group comparison with composite scores for cognitive domains

### Pattern of regional cortical thinning

**Fig 2:**
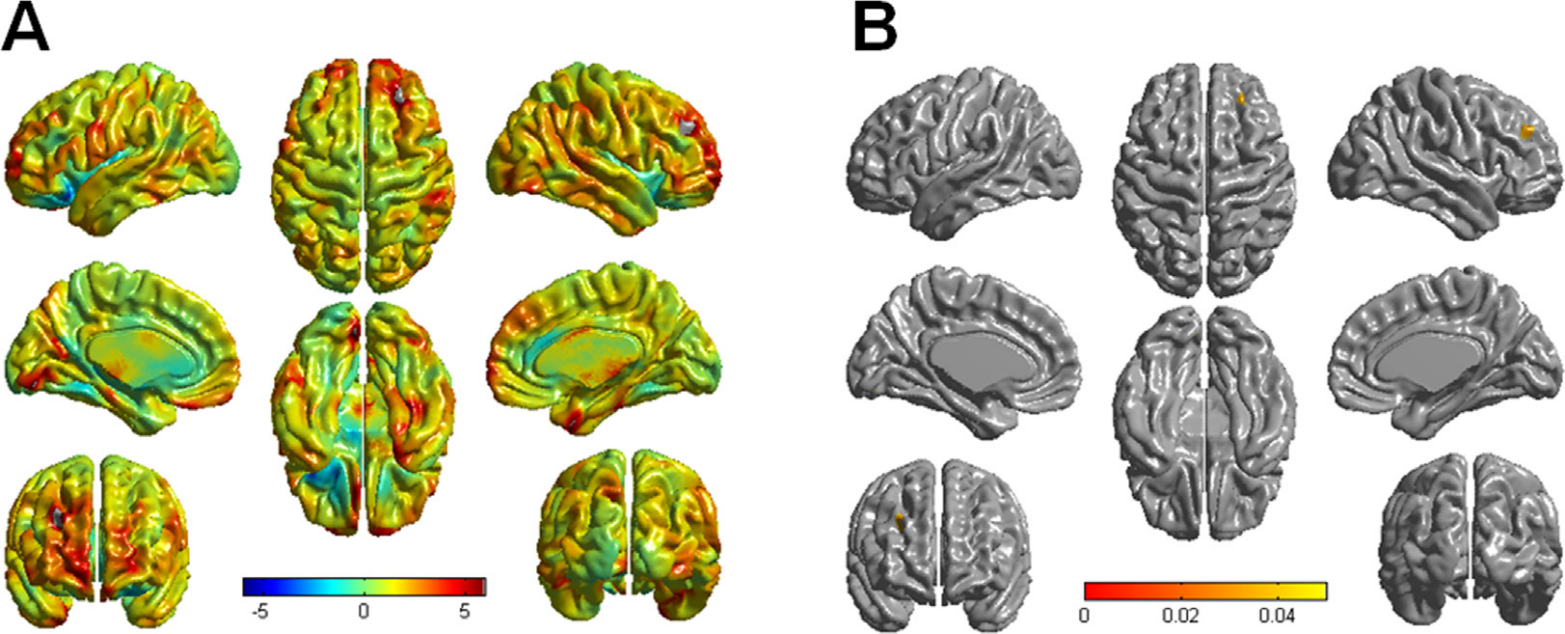
Comparison of cortical thickness in patients with Parkinson’s disease (PD) with controls. (A) The red color indicates severe differences between groups in cortical thickness. The PD-punder group tended to have more severe cortical atrophy in the bilateral dorsolateral prefrontal and orbitofrontal cortices, in the right anterior temporal cortex, in the right parietal cortex, and in the left medial occipital cortex. (B) In the corrected *t*-statistical maps of cortical thickness, only the right dorsolateral prefrontal cortex showed a statistically lower degree of cortical thickness in the PD-punder group compared with healthy controls (RFT-corrected *p* < 0.05).

PD-punders performed brain MRI within 6 months before and after diagnosis of punding. Median time from the onset of punding to brain MRI was 8 months. Significant differences between healthy controls and PD-punders in regional cortical thickness are shown in Fig 2. In Fig 2A, the red color indicates more severe differences between groups in cortical thickness. The PD-punders had a trend toward more severe cortical atrophy in the bilateral dorsolateral prefrontal cortices (DLPFC) and orbitofrontal cortices (OFC), the right anterior temporal cortex, the right parietal cortex, and the left medial occipital cortex. In the corrected *t*-statistical maps of cortical thickness (Fig 2B), PD-punders showed a significant lower degree of cortical thickness than did healthy controls in only the right DLPFC (RFT-corrected *p* < 0.05). However, clusters displaying significant difference in cortical thickness were observed neither between PD-nonpunders and control nor between PD-punders and PD-nonpunders. On correlation analysis, we found that cortical thickness was not significantly associated with punding rating scales.

**Fig 3:**
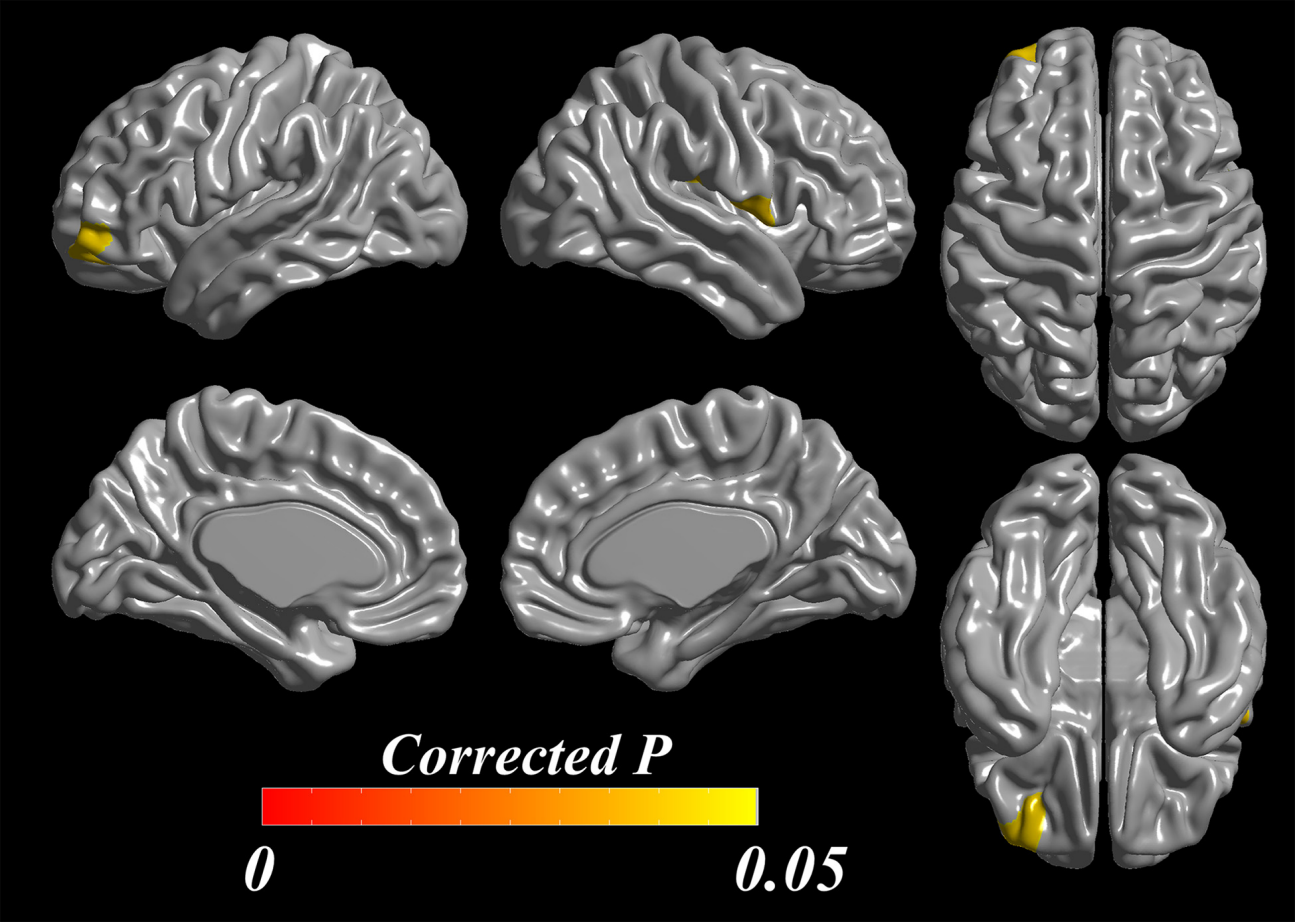
ROI-based analysis of cortical thickness in comparison of PD-punders versus PD-nonpunders. In statistical maps of cortical thickness based on frontal ROIs, the left orbitofrontal cortex (*p* = 0.042) and the right frontal operculum (*p* = 0.041) showed cortical thinning in PD-punders compared with PD-nonpunders after adjusting age, gender, PD duration, and duration of education.

In the analysis of the ROI-level differences in cortical thickness, PD-punders exhibited cortical thinning through widespread areas in frontal, temporal, parietal, and occipital lobes compared with controls. However, in PD-nonpunders group, cortical thinning area was localized in small areas in frontal and parietal lobes compared with controls. (S1 Fig). In a direct comparison between the groups, the PD-punders exhibited cortical thinning in left middle frontal cortex extending into orbitofrontal cortex (*p* = 0.042) and right frontal operculum (*p* = 0.041) compared with the PD-nonpunders (Fig 3).

## Discussion

This is the first reported study to analyze patterns of neurocognitive profiles and thinning of the cortex in non-demented PD patients with punding behavior. In the present study, we demonstrated that PD-punders performed poorly on cognitive tasks involving frontal executive functions and had regional cortical thinning in the dorsolateral prefrontal and orbitofrontal areas. These findings suggest that the DLPFC and OFC and its related frontal executive functions contribute to the development of punding behavior in patients with PD.

The reported prevalence of punding in PD patients was quite variable, in the range of 1.4–14%, depending on the questionnaires or interview tools used to screen for punding in each study. Additionally, it is quite difficult to detect punding without detailed interviews of patients and their relatives as physicians often seem to overlook the phenomenon and/or misdiagnose it. Patients can also neglect to mention the behavior or consider it rather fascinating and soothing. The present study used rigorous direct interviews conducted by neurological specialists and found that the prevalence was 5.4% in patients with PD, which is consistent with the figures reported by previous studies.[3,7,24,25] In terms of the predisposing factors for the development of punding, high daily LED, young age of onset, being male, frequent rescue doses, long PD duration, presence of dyskinesia, and disease severity have been suggested as risk factors. Similarly, our data indicated that long duration of PD and PD medication, high total daily LED, and presence of dyskinesia and ICD were significant risk factors for developing punding behavior.

Although the pathophysiology of punding in PD has not yet been established, it may be closely associated with plastic changes in the striatum linked to psychomotor stimulation and reward mechanism by means of dopaminergic medications.[5,7,26] Changes in neural plasticity cause functional alterations in dopamine transmission in the striatum, and the striatum eventually becomes sensitized to the enhanced dopamine transmission.[27,28] Because the sensitization progresses to an impaired stimuli-reward circuit, goal-directed motivation no longer triggers adequate stimulation and subsequent appropriate rewards, the behavior becomes automatic, and behavioral sensitization eventually occurs. Together with neuroadaptation and impaired reward circuits, frontal lobe function and its relation with the ventral striatum through fronto-striatal circuits has been suggested to control and modulate the dopamine-dependent induction of the repetitive stereotypic behavior.[29,30] According to the detailed neurocognitive testing we performed, the PD patients with punding tended to perform more poorly on the frontal executive function including color Stroop test than did those without punding, especially reflecting inhibitory control of execution. In the voxel-based analysis of cortical thickness, PD-punders showed significant cortical thinning in DLPFC relative to controls, whereas PD-nonpunders had cortical thickness comparable to controls. Additionally, ROI-based analysis showed that the PD-punders exhibited cortical thinning in left middle frontal cortex extending into OFC and right frontal operculum relative to the PD-nonpunders.

It is well-known that DLPFC is engaged primarily in goal setting and planning, that OFC is important in impulse control and inhibitory function, and that the medial prefrontal cortex or anterior cingulate cortex serves emotion and motivation.[31] According to many reports, the possible substrates for the development of ICDs include the ventromedial prefrontal cortex, anterior cingulate cortex, and orbitofrontal cortex.[26,32,33] Its impairment and subsequent damage to the reward circuitry, reduced control of impulsivity, and lack of motivation result in ICDs. In punding behavior, however, impairment of inhibitory control is important feature, but non-goal-directed action and aimless behavior is also a necessary condition to develop punding behavior. Although DLPFC and OFC are structurally and functionally distinctive areas each other, both areas are subdivisions of the prefrontal cortex. Many studies reported the contribution of the function of two cortices in neurological and psychological disorders.[34] The dysfunction of DLPFC can play a role in the aimless and unplanned movements and destructed OFC can contribute to impaired inhibition of action. Thus, we can postulate that prefrontal cortex including the anterior and anterolateral parts of the middle frontal gyrus may modulate the development of punding. Right frontal operculum, pars opercularis of the inferior frontal gyrus, is another candidate for the neural substrate of punding. The role of frontal operculum in non-dominant side has not yet been revealed well. Few studies reported the motor and cognitive function of the frontal operculum that it plays an important role in the suppression of response tendencies, and it also is related to hand movements.[35,36] The right frontal operculum may play as ancillary role in stereotypic behaviors in connection with prefrontal cortex. Accordingly, we suggest from the present study that the frontal cortex involving dorsolateral and orbitofrontal areas and its related cognitive domain may be a prime neuroanatomical and cognitive substrate of punding, indicating that the presence or degree of frontal dysfunction may be a predisposing factor for the development of punding behavior in patients with PD.

Many reports have demonstrated a relationship between types of DRT and DRT-related complications.[4] Newer dopamine agonists, particularly pramipexole, acting mainly on D3 subtype receptors, have been reported to be powerful ICD-inducing agents according to a few studies. Some reports insist that dopamine agonists are relevant to punding behavior,[37] or that levodopa contributes proximately to the development of punding.[5] In the present study, PD-punders tended to have a higher levodopa LEDs than did PD-nonpunders, whereas dopamine agonist LEDs did not differ between groups. We think that punding seems to be related with the dosage of levodopa medication rather than dopamine agonists. However, a larger sample size of the PD-punder group is needed to assess the specific dopamine receptor(s) responsible for punding behavior in patients with PD.

Several limitations of our study need to be addressed. First, the number of cases of PD patients with punding was too small. Large numbers of cases are needed to draw firm conclusions about the exact cognitive profile and neuroanatomical substrate involved in punding. Nevertheless, we recruited punders via a direct interview with trained neurologists, and misdiagnosis or underestimation of punding seems unlikely. Second, we could not get a result of difference in direct comparison of cortical thickness between PD-punders and PD-nonpunders in the vertex-based analysis of whole cerebral cortex. Further studies with large cases are necessary to compare the frontal cortical atrophy directly between two groups. Third, the presence of ICD and dyskinesia in PD is frequently accompanied by punding behavior. The results of the present study may not exactly reflect the effects of punding alone. Fourth, PD-punders group completed neuropsychological tests and underwent brain MRI within 6 months before and after diagnosis of punding. Examinations scheduled at fixed intervals relative to the appearance of punding would be the best approach to making exact comparisons between two groups. Fifth, PD-punders did not show a significant difference in all cognitive items after correction for multiple comparisons, and there was a tendency of declined cognition in frontal executive function. Therefore, the results neurocognitive profiles should be interpreted cautiously.

In summary, we demonstrated that PD patients with punding had a tendency of cognitive deficits in the frontal executive functions, and showed more extensive cortical atrophy in the dorsolateral prefrontal and orbitofrontal areas. These data indicate that prefrontal modulation may be an essential component in the development of punding behavior in patients with PD.

## Supporting Information

S1 Fig ROI-based analysis of cortical thickness in comparison of PD-punders and PD-nonpunders with controls. (A) The patients with PD without punding exhibited cortical thinning through widespread areas including frontal, temporal, parietal, and occipital lobes compared with controls. The areas are composed of the bilateral dorsolateral superior frontal gyri, left orbital superior frontal gyrus, right lateral middle frontal gyrus, left orbital middle frontal gyrus, bilateral rolandic operculum, bilateral supplementary motor areas, right medial and medial orbital superior frontal gyrus, bilateral parahippocampal gyri, left cuneus, right lingual gyrus, left middle occipital area, bilateral fusiform gyri, bilateral inferior parietal lobules, right supramarginal gyrus, left angular gyrus, bilateral superior temporal gyri, right superior temporal pole, bilateral middle temporal gyri, right middle temporal pole, and left inferior temporal gyrus. (B) The areas of cortical thinning in patients with PD with punding relative to controls were localized in small regions in frontal and parietal lobes. The areas consists of left rolandic operculum and bilateral supramarginal gyri.
(TIF)
