# Supplementary figures and images for: Patterns of Neuropsychological Profile and Cortical Thinning in Parkinson’s Disease with Punding
Source: PLoS One. 2015 Jul 28;10(7):e0134468. doi: 10.1371/journal.pone.0134468 (PMC4517876; doi:10.1371/journal.pone.0134468)

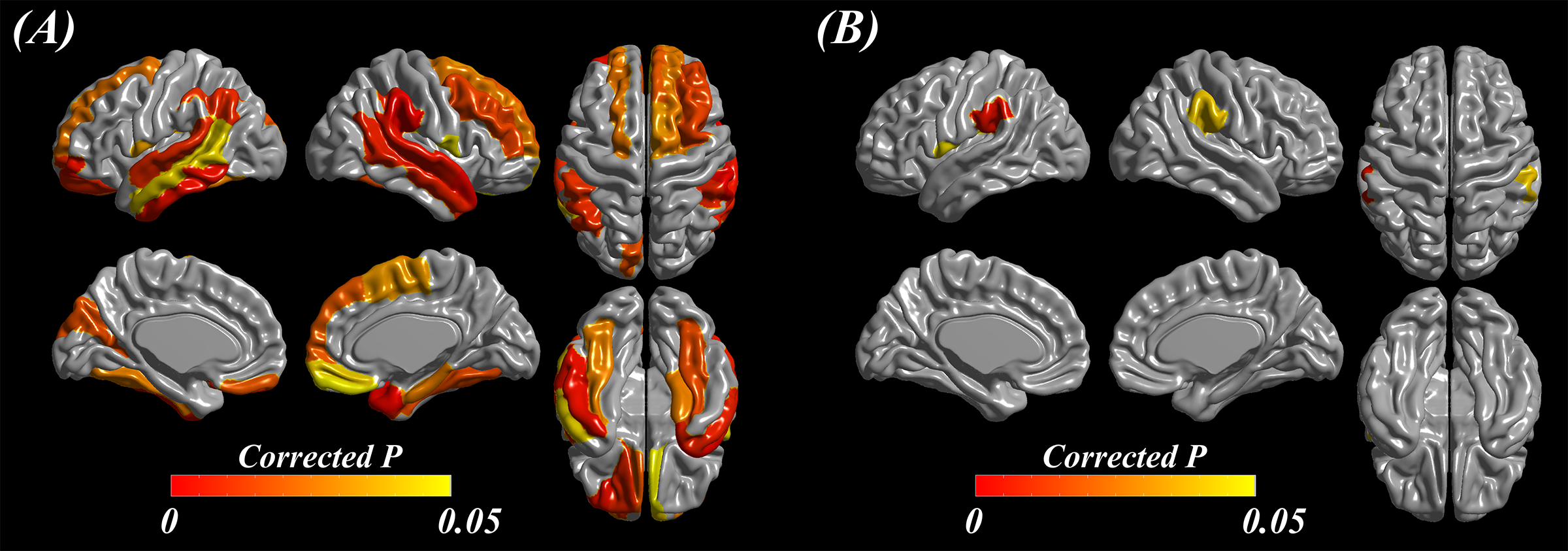

Supplement: S1 Fig — (A) The patients with PD without punding exhibited cortical thinning through widespread areas including frontal, temporal, parietal, and occipital lobes compared with controls. The areas are composed of the bilateral dorsolateral superior frontal gyri, left orbital superior frontal gyrus, right lateral middle frontal gyrus, left orbital middle frontal gyrus, bilateral rolandic operculum, bilateral supplementary motor areas, right medial and medial orbital superior frontal gyrus, bilateral parahippocampal gyri, left cuneus, right lingual gyrus, left middle occipital area, bilateral fusiform gyri, bilateral inferior parietal lobules, right supramarginal gyrus, left angular gyrus, bilateral superior temporal gyri, right superior temporal pole, bilateral middle temporal gyri, right middle temporal pole, and left inferior temporal gyrus. (B) The areas of cortical thinning in patients with PD with punding relative to controls were localized in small regions in frontal and parietal lobes. The areas consists of left rolandic operculum and bilateral supramarginal gyri. (TIF) [file pone.0134468.s001.tif]
